# Prevalence of Chronic Kidney Disease in Patients with Diabetes in Extremadura (Spain) during the Years 2012, 2013 and 2014: An Observational Study

**DOI:** 10.3390/jcm10132886

**Published:** 2021-06-29

**Authors:** Leandro Fernández-Fernández, Alfonso Barquilla-García, Javier Sánchez-Vega, José Carlos Risco-Solanilla, Félix Suárez-González, Francisco Buitrago

**Affiliations:** 1 Servicio Extremeño de Salud, Centro de Salud de Zafra, 06300 Badajoz, Spain; leferfer@gmail.com; 2 Servicio Extremeño de Salud, Centro de Salud de Trujillo, 10200 Cáceres, Spain; alfonso.barquilla@gmail.com; 3 Servicio Extremeño de Salud, Centro de Salud de Losar de la Vera, 10460 Cáceres, Spain; javiersanchezvega55@gmail.com; 4 Servicio Extremeño de Salud, Centro de Salud de Villarta de los Montes, 06678 Villarta de los Montes, Spain; jcarlos.riscosolanilla@gmail.com; 5 Servicio Extremeño de Salud, Centro de Salud de “San Roque”, 06008 Badajoz, Spain; fsuarezgenator3@gmail.com; 6 Servicio Extremeño de Salud, Centro de Salud Universitario “La Paz”, Departamento de Ciencias Biomédicas, Universidad de Extremadura, 06007 Badajoz, Spain

**Keywords:** chronic kidney disease, renal impairment, albuminuria, type 2 diabetes mellitus, primary health care

## Abstract

Diabetes mellitus (DM) is one of the leading causes of chronic kidney disease (CKD). We analyzed the prevalence of CKD in the population with diabetes in Extremadura (Spain). retrospective observational study was carried in the diabetic population attended in the Extremadura Health System in 2012–2014. A total of 38,253 patients, ≥18 years old were included. Estimated glomerular filtration rate (eGFR) was calculated using the CKD Epidemiology Collaboration equation. CKD was defined as follow: an eGFR <60 mL/min/1.73 m^2^ in a time period ≥ of three months or the presence of renal damage, with or without reduced eGFR, if the urine albumin-creatinine ratio (UACR) was ≥30 mg/g, also in a time period ≥ of three months. The prevalence rate of CKD was 25.3% (27.6% in women; 23.0% in men) and increases with age (34.0% in ≥65 years-olds). 24.9% of patients with CKD were in the very-high risk category for cardiovascular events (6.3% of the diabetic population). If CKD were diagnosed without requiring sustained eGFR <60 mL/min/1.73 m^2^ and/or sustained UACR ≥30 mg/g (as it is frequently found in the literature) this would overestimate the prevalence of CKD by 23%.

## 1. Introduction

Chronic kidney disease (CKD) and type 2 diabetes mellitus (T2DM) are highly prevalent chronic diseases, and both represent major public health problems. The estimated prevalence of CKD in the world population over 30 years of age is 7.2% [1], and in Spain is 6.8% [2]. The progressive aging of the population and the increase in diseases such as diabetes, obesity and high blood pressure could double the incidence of CKD in the coming decades [3,4]. The incidence and prevalence of diabetes mellitus (DM) has increased worldwide, mainly due to T2DM. This global increase in the number of diabetic patients has had a major impact on the development of CKD, as DM is the main cause of CKD, accounting for approximately 50% of cases [5].

Patients with DM and renal impairment are a group of patients with higher morbidity and mortality, mainly cardiovascular, in addition to having a higher risk of hypoglycemia. The incidence of cardiovascular events in patients with CKD is very high and similar to that of patients with coronary heart disease. Indeed, it is accepted that CKD behaves as an equivalent of coronary risk, and therefore CKD patients are candidates for secondary cardiovascular prevention measures [6]. On the other hand, according to studies in different countries, the prevalence of CKD in diabetic patients ranges from 20–40% [7,8,9], and in Spain it is estimated as 27.9% [10], although the diagnostic criteria used are not homogeneous.

Given this background, the objective of this study was to establish the prevalence of CKD in the diabetic population of Extremadura (Spain), using the diagnostic criteria proposed in the KDIGO 2012 guidelines (Kidney Disease Improving Global Outcome) [11].

## 2. Materials and Methods

We performed an observational, retrospective, prevalence study, with the information gathered, during 2012, 2013 and 2014, in two databases from the Extremadura Health System: one about medication dispensed in pharmacies, and another about clinical analyses (CORNALVO). Patients, ≥18 years old with DM criteria were identified as previously reported [12] (90,709; average age 68.3 ± 14.1 years; 50.2% women). Among them, in the three-year period studied, 76,408 patients (mean age 68.2 ± 14.0 years; 50.3% women) had at least one serum creatinine determination, and 46,129 patients (mean age 66.9 ± 13.9 years; 49.8% women) had at least one creatinine and albumin determination in urine. Only 38,253 individuals (mean age 65.9 ± 13.9 years; 49.3% women) had the necessary data to apply the KDIGO definition of CKD, i.e., data for both the glomerular filtration rate (eGFR) and the urine albumin/creatinine ratio (UACR) on at least 2 occasions ≥ 3 months apart [11]. And so, only for these patients could an actual (true) CKD diagnosis be made. However, with some frequency the estimation of the prevalence of CKD is made with a single eGFR determination [7,8,10,13,14]. For comparative purposes, and just to estimate the effect of this less strict diagnostic criterion in the estimated prevalence of CKD, patients for whom only one serum creatinine determination was available were analyzed separately.

The glomerular filtration rate (eGFR) was estimated using the equation developed by the Chronic Kidney Disease Epidemiology Collaboration (CKD-EPI) [15]. The urine albumin/creatinine ratio (UACR) was used as a measure of kidney damage.

Any patient with DM was considered to have CKD if he had, at his last analytical control during the study period, an eGFR value below 60 mL/min/1.73 m^2^ and/or an UACR ≥30 mg/g, confirmed in a previous determination separated by at least 3 months [11,16]. The eGFR was stratified into 6 categories (stages) (G1, G2, G3a, G3b, G4 and G5) and the UACR into 3 categories (A1, A2 and A3), according to the recommendations of the Kidney Disease Improving Global Outcomes (KDIGO) guidelines [11].

Extremadura is one of Spain’s Autonomous Communities and, in 2013, had a total population of 1,104,004 inhabitants (548,054 males) [17]. 95.3% of this population uses the publicly funded Extremadura Health System (Servicio Extremeño de Salud—SES), to which the results of this study refer. The remaining 4.7% of the population of Extremadura uses other health service providers (European Health Survey, 2014) [18].

Since anonymised electronic records were used as a data source, written informed consent was not required.

Data processing and analysis were performed using SPSS 22.0 for Windows. Results were stratified by age group and by sex. In bivariate analyses, a *t*-test for independent samples was used for quantitative variables and a Chi-squared test for categorical variables.

## 3. Results

The prevalence rate of CKD in patients with DM and data for both eGFR and UACR on at least 2 occasions ≥ 3 months apart (38,253 patients; mean age 65.9 years; 49.3% women) was 25.3% (27.6% in women and 23.0% in men, *p* < 0.001). Slightly more than half of that percentage (12.8%) corresponded to stage 3 CKD (eGFR between 59–30 mL/min/1.73 m^2^). The higher prevalence of CKD was found in the group ≥65 years old (34.0%; 35.4% in women and 32.1% in men), followed by the group 40–64 years (13.6%) (Table 1).

Table 2 shows that 6.3% of those 38,253 have a very-high risk of fatal cardiovascular events and progression of impaired renal function (the 2406 diabetics resulting from the sum of the groups G5, G4, G3bA2, G3bA3, and G3aA3). That is to say, 24.9% of 9674 diabetics with CKD (Table 1) are in the category of very-high cardiovascular risk.

The distribution of the diabetic population with a single eGFR or UACR test is shown in Table 3. In this population (76,408 patients; 68.2 years; 50.3% women) there is a 31.0% of patients (34.8% in female and 27.3% in male, *p* < 0.001), with a single eGFR <60 mL/min/1.73 m^2^ and/or a single UACR ≥ 30 mg/g. Accordingly, if this were accepted as a true diagnose of CKD, the prevalence rate would be overestimated by 23% (by 26% in women, and by 19% in men).

## 4. Discussion

### 4.1. Summary of Main Findings

The estimated prevalence of CKD in the diabetic population of Extremadura ≥18 years of age is 25.3%, with slightly more than half of that percentage (12.8%) corresponding to stage 3 CKD (eGFR between 59–30 mL/min/1.73 m^2^) and 11.7% to patients with an A2 category of kidney damage (UACR between 30–300 mg/g). The prevalence of CKD increases with age, so that 34.0% of those ≥ 65 years of age have stage 3 or higher CKD. 24.9% of patients with CKD are included in the very-high risk category for cardiovascular events (6.3% of the diabetic population).

### 4.2. Strengths and Limitations of This Study

The main strengths of this study are that it analyses the information referring to virtually the entire population of Extremadura (95.3%), users of the Public Health System, and that it uses as diagnostic criteria for CKD those recommended by the National Kidney Foundation K/DOQI clinical practice guidelines [16], which require confirmation of a decreased eGFR or the presence of kidney damage in another determination, separated by at least 3 months.

This study also has limitations. The identification of patients with diabetes was carried out with diagnostic criteria based on hypoglycemic drug use and hemoglobin A_1C_ values and not through the review of patients’ medical history [12]. This strategy has the advantage of identifying a large number of patients with hidden diabetes, but the disadvantage is that the adopted diagnostic criteria will also include as diabetic patients a small number of patients who might have a prescription for antidiabetic drugs for an indication other than diabetes, for example, polycystic ovarian syndrome (metformin), obesity (metformin and incretin analogues), or gestational diabetes (metformin, insulin) [12]. Moreover, the databases used for the study do not contain data about possible urine sediment abnormalities, imaging abnormalities, kidney transplantation or pathologic abnormalities. Therefore, it is not possible to ascertain how many patients underwent a renal biopsy or whether they were diagnosed diabetic nephropathy or other pathologies. On the other hand, our results correspond to the sub-population with complete data and it is probable that the individuals with laboratory tests available are qualitatively different from those without test done, and thus representing a selection bias.

### 4.3. Comparison with Existing Literature

The prevalence of CKD in the diabetic population in our study (25.3%) is in the prevalence range found (23.0–27.9%) in our country [10,13,14]. However, in these studies the definition of CKD is made with a single determination of eGFR, without strictly complying with the recommendation of confirming the decrease in the eGFR or the manifestation of kidney damage in another determination, separated by at least three months in time [11,16].

The prevalence of CKD in our study is also in the range of 20–40% reported for the prevalence of CKD in the diabetic population of other countries [19], although again in these studies the definition of CKD is also usually made with a single eGFR determination [7,8]. That way of estimating the prevalence overestimates the prevalence of CKD in the diabetic population and does not accurately reflect the true prevalence rate of CKD, since a single decreased eGFR value or increased UACR, as manifestation of renal damage, may correspond to transient situations rather than chronic renal functional impairment, as required by the definition of CKD [11]. In fact, if in our study we were to consider a single value of eGFR <60 mL/min/1.73 m^2^ and/or a single UACR ≥ 30 mg/g as the definition of CKD, the prevalence would be 31.0% (Table 3). However, not fully applying the criteria that define a certain disease by consensus, such as CKD, is an artificial way of increasing its prevalence. For example, in our specific case, the prevalence rate of CKD would be overestimated by 23% (by 26% in women, and by 19% in men) if it were diagnosed without a sustained eGFR <60 mL/min/1.73 m^2^ and/or a sustained UACR ≥ 30 mg/g.

Finally, it should be noted that in the three-year period analyzed, only 84.2% of the diabetic population had a serum creatinine determination that allowed the calculation of eGFR, and only 50.9% had an UACR determination made. We do not know the reasons why 15.8% and 49.1% of diabetic patients, respectively, lacked these determinations, when the recommendations of the clinical guidelines are to make them at least once a year [19]. The existence of a subgroup of the population in whom UACR is not measured, also needs to be emphasized from a population health perspective, given that diabetes is a condition characterized by glomerular hyperfiltration and albuminuria is a marker of renal damage, that precedes a fall in GFR. Accordingly, these results also force us to review the quality of care provided to patients with DM in Extremadura (Spain) [12].

### 4.4. Implications for Future Research and for Clinical Practice

CKD is associated with poor clinical outcomes, including an increased risk of all-cause and cardiovascular mortality, as well as adverse economic and social effects. Slowing the development and progression of CKD remains an unmet clinical need in patients with T2DM. Actually, the rapidly emerging evidence favoring SGLT2 inhibition means that high-risk CKD patients need to be identified early in the course of their disease, especially those with proteinuria and preserved kidney function in whom there is now an opportunity to slow down the trajectory of GFR decline [20].

In summary, our study reveals a prevalence of CKD of 25.3% in the diabetic population of Extremadura (Spain), accepting the criteria defined in the K/DOQI clinical practice guidelines of the National Kidney Foundation. On the other hand, the high percentage of patients without eGFR and UACR determinations is in line with the topic of clinical/therapeutic inertia which concerns both nephrologists and diabetologists, since glomerular hyperfiltration and albuminuria are often the first marker of a progression towards CKD [21]. An early recognition of risk factors for CKD progression can be decisive in decreasing morbidity and mortality, and a timely nephrological evaluation can also guarantee adequate information to choose the right renal replacement therapy at the right time in case of renal impairment progression. Therefore, it is necessary to re-evaluate the quality of care received by the diabetic population in Extremadura (Spain) [12]. A systematic determination of eGFR and UACR would contribute to earlier diagnosis of CKD, thus allowing intervention in the early stages of the disease where treatment is more effective.

## Figures and Tables

**Table 1:** Distribution of the diabetic population with CKD diagnosed by a sustained eGFR <60 mL/min/1.73 m^2^ and a sustained UACR ≥ 30 mg/g.

**Diabetic Population**	**18–39 Years** **(*n* = 1790)**	**40–64 Years** **(*n* = 13,976)**	**≥65 Years** **(*n* = 22,487)**	**Total** **(*n* = 38,253)**	***p* Value**
CKD, *n* (%)	125 (7.0)	1907 (13.6)	7642 (34.0)	9674 (25.3)	<0.001
Age, mean (SD), years	34.1 (4.6)	56.3 (6.1)	77.7 (6.6)	73.0 (11.6)	<0.001

**Women**	**18–39 Years** **(*n* = 783)**	**40–64 Years** **(*n* = 5270)**	**≥65 Years** **(*n* = 12,824)**	**Total** **(*n* = 18,877)**	***p* Value**
CKD, *n* (%)	53 (6.8)	621 (11.8)	4543 (35.4)	5217 (27.6)	<0.001
Age, mean (SD), years	33.9 (5.2)	56.6 (6.2)	78.7 (6.4)	75.6 (10.5)	<0.001

**Men**	**18–39 Years** **(*n* = 1007)**	**40–64 Years** **(*n* = 8706)**	**≥65 Years** **(*n* = 9663)**	**Total** **(*n* = 19,376)**	***p* Value**
CKD, *n* (%)	72 (7.2)	1286 (21.7)	3099 (32.1)	4457 (23.0)	<0.001
Age, mean (SD), years	34.2 (4.1)	56.2 (6.0)	76.3 (6.5)	69.9 (12.0)	<0.001

**Table 2:** Estimation of risk levels according to eGFR and UACR data of the 38,253 patients for which two eGFR and UACR values were available separated by at least 3 months. The colors indicate the risk of global mortality, cardiovascular mortality, kidney failure treated with dialysis or transplantation, acute kidney failure and progression of kidney disease [11,16]: **green**, low risk (if no other markers of kidney disease, no CKD); **yellow**, moderately increased risk; **orange**, high risk; **red**, very high risk.

		A1 * (UACR: <30 mg/g)	A2 (UACR: 30–300 mg/g)	A3 (UACR: >300 mg/g)	Total
G1 * (eGFR: >90 mL/min/1.73 m^2^)	Women, *n* (%)		417 (2.21)	64 (0.34)	6377 (33.78)
	Men, *n* (%)		735 (3.79)	106 (0.55)	8743 (45.12)
	Total, *n* (%)		1152 (3.01)	170 (0.44)	15,120 (39.53)
G2 (eGFR: 89–60 mL/min/1.73 m^2^)	Women, *n* (%)	7764 (41.13)	785 (4.16)	115 (0.61)	8664 (45.90)
	Men, *n (%)*	7017 (36.21)	955 (4.93)	235 (1.21)	8207 (42.36)
	Total, *n* (%)	14,781 (38.64)	1740 (4.55)	350 (0.91)	16,871 (44.10)
G3a (eGFR: 59–45 mL/min/1.73 m^2^)	Women, *n* (%)	1222 (6.47)	272 (1.44)	57 (0.30)	1551 (8.22)
	Men, *n* (%)	641 (3.31)	237 (1.22)	84 (0.43)	962 (4.96)
	Total, *n* (%)	1863 (4.87)	509 (1.33)	141 (0.37)	2513 (6.57)
G3b (eGFR: 44–30 mL/min/1.73 m^2^)	Women, *n* (%)	999 (5.29)	368 (1.95)	110 (0.58)	1477 (7.82)
	Men, *n* (%)	485 (2.50)	282 (1.46)	145 (0.75)	912 (4.71)
	Total, *n* (%)	1484 (3.88)	650 (1.70)	255 (0.67)	2389 (6.25)
G4 (eGFR: 29–15 mL/min/1.73 m^2^)	Women, *n (%)*	362 (1.92)	222 (1.18)	109 (0.58)	693 (3.67)
	Men, *n* (%)	121 (0.62)	144 (0.74)	153 (0.79)	418 (2.16)
	Total, *n* (%)	483 (1.26)	366 (0.96)	262 (0.68)	1111 (2.90)
G5 (eGFR: < 15 mL/min/1.73 m^2^)	Women, *n* (%)	24 (0.13)	24 (0.13)	67 (0.35)	115 (0.61)
	Men, *n* (%)	11 (0.06)	22 (0.11)	101 (0.52)	134 (0.69)
	Total, *n* (%)	35 (0.09)	46 (0.12)	168 (0.44)	249 (0.65)
Total	Women, *n* (%)	16,267 (86.17)	2088 (11.06)	522 (2.77)	18,877 (100)
	Men, *n (%)*	16,177 (83.49)	2375 (12.26)	824 (4.25)	19,376 (100)
	Total, *n* (%)	32,444 (84.81)	4463 (11.67)	1346 (3.52)	38,253 (100)

UACR = Urine albumin/creatinine ratio. eGFR = estimated glomerular filtration rate.* The G1A1 cells are empty because for the 13,798 patients (5896 women and 7902 men) fitting these eGFR and UACR categories, the databases used for the study do not contain data about possible urine sediment abnormalities, imaging abnormalities, renal biopsies, pathologic abnormalities or kidney transplantation.

**Table 3:** Distribution of the diabetic population that underwent only a single test of eGFR and/or UACR.

**Diabetic Population**	**18–39 Years** **(*n* = 2798)**	**40–64 Years** **(*n* = 24,514)**	**≥65 Years** **(*n* = 49,096)**	**Total** **(*n* = 76,408)**	***p* Value**
Patients with a single eGFR <60 mL/min/1.73 m^2^ and/or UACR ≥ 30 mg/g, *n* (%).	241 (8.6)	3592 (14.7)	19,879 (40.7)	23,712 (31.0)	<0.001
Age, mean (SD), years	33.8 (4.7)	56.6 (6.0)	79.2 (7.0)	75.3 (11.4)	<0.001

**Women**	**18–39 Years** **(*n* = 1269)**	**40–64 Years** **(*n* = 9174)**	**≥65 Years** **(*n* = 27,964)**	**Total** **(*n* = 38,407)**	***p* Value**
Patients with a single eGFR <60 mL/min/1.73 m^2^ and/or UACR ≥ 30 mg/g, *n* (%).	100 (7.9)	1253 (13.7)	11,998 (42.9)	13,351 (34.8)	<0.001
Age, mean (SD), years	33.6 (5.3)	56.8 (6.1)	80.1 (6.8)	77.6 (10.3)	<0.001

**Men**	**18–39 Years** **(*n* = 1529)**	**40–64 Years** **(*n* = 15,340)**	**≥65 Years** **(*n* = 21,132)**	**Total** **(*n* = 38,001)**	***p* Value**
Patients with a single eGFR <60 mL/min/1.73 m^2^ and/or UACR ≥ 30 mg/g, *n* (%).	141 (9.2)	2339 (15.3)	7881 (37.3)	10,361 (27.3)	<0.001
Age, mean (SD), years	34.0 (4.1)	56.5 (6.0)	77.7 (6.9)	72.3 (12.0)	<0.001

## Data Availability

The databases mentioned in Materials and Methods, from where the reported results were extracted, are owned by the Servicio Extremeño de Salud (Extremadura Health System, Mérida, Spain). These data were used with permission from the Managing Director of Health and Socio-sanitary Planning, Training and Quality, from the Ministry of Health and Social Policies of the Government of Extremadura.
